# Use of a clinical PET/MR scanner for preclinical research with first results

**DOI:** 10.1186/2197-7364-1-S1-A88

**Published:** 2014-07-29

**Authors:** Karthik Chary, Jarmo Teuho, Jenni Virta, Hannu Sipilä, Virva Saunavaara, Anne Roivainen, Mika Teräs

**Affiliations:** Turku PET Centre, Turku University Hospital, Turku, Finland

This study was performed to evaluate the feasibility of preclinical imaging in a clinical PET/MR system. Preliminary sequences were evaluated for establishing preclinical protocols for rat brain and rabbit knee. Rats were placed in a stereotactic holder, allowing a 30 minute scan time before re-administration of anesthesia. In-house developed warm-water heating system was used to maintain the body temperature at 37.5°C, monitored using an MR-compatible rectal probe. Brain imaging was performed with a dedicated 4 channel phased array receive coil (RAPID Biomedical GmbH, Germany). High resolution coronal images were acquired using conventional T1-SE (0.30x0.30x1.2mm) and T2-TSE (0.23x0.23x0.7mm) with a total scan time of 30 min. PET/MR imaging was performed on two white rabbits. The rabbits were imaged in a custom wooden holder. PET/MR protocol had a total duration of 45 minutes. No external heating was used. MR protocol consisted of anatomical T1, T2 and PDW of the knees, using a SENSE Flex-S coil. MR attenuation correction (MRAC) was acquired with 3D T1-FFE using three-class segmentation. A dynamic 30 minute PET acquisition was started on injection of 33.8MBq of Ga-68. Animal coils enabled high resolution images to be acquired in reasonable acquisition time with regards to animal handling and anesthesia. T1 and T2 images provided good differentiation of anatomy in the rat brain with high contrast. T1, T2 and PDW images of the rabbit knee had high resolution and differentiation of anatomical structures. MRAC was able to distinguish the knees and the body contour. Image fusion of PET and MR was able to localize the infection, which was confirmed by a physician. Pre-clinical imaging with the Ingenuity TF was deemed feasible, although PET imaging is limited by the resolution of the scanner. The preliminary sequences were successfully implemented for future studies on the Ingenuity TF.

